# Patient’s Radiation Exposure in Coronary Angiography and Angioplasty: The Impact of Different Projections

**DOI:** 10.15171/jcvtr.2014.020

**Published:** 2014-12-30

**Authors:** Alireza Farajollahi, Atena Rahimi, Ebrahim Khayati Shal, Samad Ghaffari, Morteza Ghojazadeh, Arezou Tajlil, Naser Aslanabadi

**Affiliations:** ^1^Medical Education Research Center, Department of Medical Physics, Faculty of Medicine, Tabriz University of Medical Sciences, Tabriz, Iran; ^2^Medical Physics Department, Faculty of Medicine, Tabriz University of Medical Science, Tabriz, Iran; ^3^Urmia University of Medical Science, Urmia, Iran; ^4^Cardiovascular Research Center, Tabriz University of Medical Science, Tabriz, Iran; ^5^Liver and Gastrointestinal Disease Research Center, Tabriz University of Medical Sciences, Tabriz, Iran

**Keywords:** Radiation, Interventional, Angiography, Angioplasty

## Abstract

***Introduction:*** We aimed to determine angiography projections with lower Dose Area Product (DAP) rate by measuring the mean DAP and fluoroscopy times in coronary angiography (CAG) and percutaneous coronary intervention (PCI) and calculating DAP rate in different projections.

***Methods:*** DAP and fluoroscopy times were measured in all employed projections in real-time in 75 patients who underwent CAG or PCI by a single cardiologist in Madani Cardiovascular University Hospital (45 in CAG group and 30 in PCI group). DAP rate was calculated in both groups and in all projections. The projections with highest and lowest DAP rate were determined.

***Results:*** Mean DAP was 436.73±315.85 dGy×cm^2^ in CAG group and 643.26±359.58 dGy×cm^2^ in PCI group. The projection 40° LAO/0° had the highest DAP rate in CAG group (28.98 dGy×cm^2^/ sec) and it was highest in 20° RAO/30° CR in PCI group (29.83 dGy×cm^2^/sec). The latter projection was also the most employed projection in PCI group.

***Conclusion:*** The amount of radiation dose in this study is in consistent with the previous reports. Specific angiographic projections expose patients to significantly higher radiation and they should be avoided and replaced by less irradiating projections whenever possible.

## Introduction

Invasive Coronary Angiography (CAG) is the most reliable method for identifying coronary artery stenosis in patients with suspected coronary artery disease (CAD). In addition to its diagnostic rule, the information gained throughout CAG, is commonly used for determining the most appropriate management of the patient.^1^ If the patient with ischemic CAD meets the criteria for percutaneous coronary intervention (PCI), coronary stents can be implemented at the same time or in another session, alternatively.^2^ The clinical benefits of coronary angiography and the recent technological advances in angiography systems, have expanded its’ usage.^1^ On the other hand, CAD is a leading cause of mortality globally and exclusion of CAD in suspected patients is of cardinal importance.^3^ Growing number of patients undergoing CAG and PCI gives rise to some concerns, regarding potential acute and long-term side effects of this procedure.^4^

CAG and PCI expose patients to a considerable amount of X-ray radiation during fluoroscopy.^4,5^ Increasing use of radiation in medical imaging and procedures has currently made medical radiation the leading source of man-made radiation exposure in population.^4^ In a report by Bedetti el al., arteriography and interventional cardiology constituted only 12% of all radiological examinations in cardiac patients, but they accounted for 48% of average dose per patient.^4,5^ Although a single CAG may induce a small radiation risk but due to repetition of the procedure in many patients, the cumulative effective dose of multiple procedures should be considered in each patient.^5,6^

The hazardous effects of ionizing radiation can be categorized to deterministic and stochastic. Deterministic effects develop above specific thresholds of absorbed dose to a particular tissue.^7^ Skin erythema, epilation, hyperpigmentation and even direct cardiac toxicity are all the examples of this type of effects. Stochastic effects of radiation lead to a damage that may end in a malignancy, generally at a much later time.^8,9^

In both CAG and PCI, patient radiation exposure can be influenced by some factors such as patient obesity, the complexity of the procedure, and tube angulations.^10^ Although using a wide range of tube angulations is possible with current equipment, most cardiologists prefer to use the predefined standard projections. However these projections may expose patients to higher levels of x-ray radiation without giving any further information in comparison to the less irradiating projections.^10,11^

Regarding these facts, achieving the practicable minimum radiation dose has to be a principal concern during CAG and PCI.^12^ Various studies have reported different radiation dose for CAG, and this value can even vary among cardiologists, using the same technology.^13-15^ On the other hand, projections, which are used for viewing the coronary arteries, may expose patients to different radiation dose.^16,17^ Therefore, measuring the radiation dose in different projections may give us an insight to choose the ones with lower radiation dose.

In this regard, we conducted a prospective study to measure the mean Dose Area Product (DAP) and mean DAP rate of CAG and PCI in real-time. The data were presented for all tube angulations that were employed by an experienced cardiologist in the clinical setting. The measured DAP and calculated DAP rate were compared to the findings of other studies and the projections with the highest and lowest radiation dose were determined, which may help cardiologists to choose the best set of feasible projections in each patient.

## Materials and methods

From June 2013 to August 2013, consecutive patients who underwent CAG or PCI in Madani Cardiovascular University hospital by an experienced academic cardiologist were entered in this study. A total number of 75 patients were entered in the study. Among the eligible cases, 45 underwent CAG and another 30 cases underwent PCI, based on current guidelines. Patients with aortic stenosis, a prior history of revascularization procedure by coronary artery bypass grafting and also an earlier pacemaker implantation, as well as patients with simultaneous right heart catheterization or aortography were excluded from the study. In all patients, right femoral artery was accessed without difficulty, and all procedures were uncomplicated.

A digital single-plane Shimadzu angiography unit was used in all studied procedures. An integrated DAP-meter ionization chamber of the angiography unit, placed beyond the X-ray collimators, was used to measure DAP during fluoroscopy. To check the consistency and accuracy of the machine exposure factors (tube voltage, exposure time, dose and dose rate) Diavolt and Diadose quality control kit (PTW-Freiburg, Germany) was used.

Measured fluoroscopy DAP values in units of (dGy×cm^2^) and the corresponding fluoroscopy times were recorded for each case in all used angles. DAP rate was calculated by dividing DAP of an angulation by the time it was employed and it was stated in unites of dGy×cm^2^/sec.

### Statistical Analysis

Statistical software SPSS (ver. 20 for Windows) was used for data analysis. Continuous variables were presented as the Mean ± Standard deviation and categorical variables were reported as Frequencies and Percentages.

## Results

The study population constituted 75 patients, of whom 45 underwent diagnostic coronary angiography, and the remainder underwent PCI. Sixty-six percent of patients in CAG group and 70% of patients in PCI group were male. The mean age of patients in CAG group was 58±10 years. The mean fluoroscopy time was 187±139 seconds. The range of projections were from 50° Right Anterior Oblique (RAO) to the 50° Left Anterior Oblique (LAO) and from 40° cranial (CR) to 40° caudal (CA).

The mean age of patients in PCI group was 56±9 years. The mean fluoroscopy time was 489±344 seconds. The range of projections in PCI were from 50° RAO to 60° LAO and from 40° cranial to 40° caudal.

### Mean DAP

### Mean DAP in CAG

**Figure 1:**
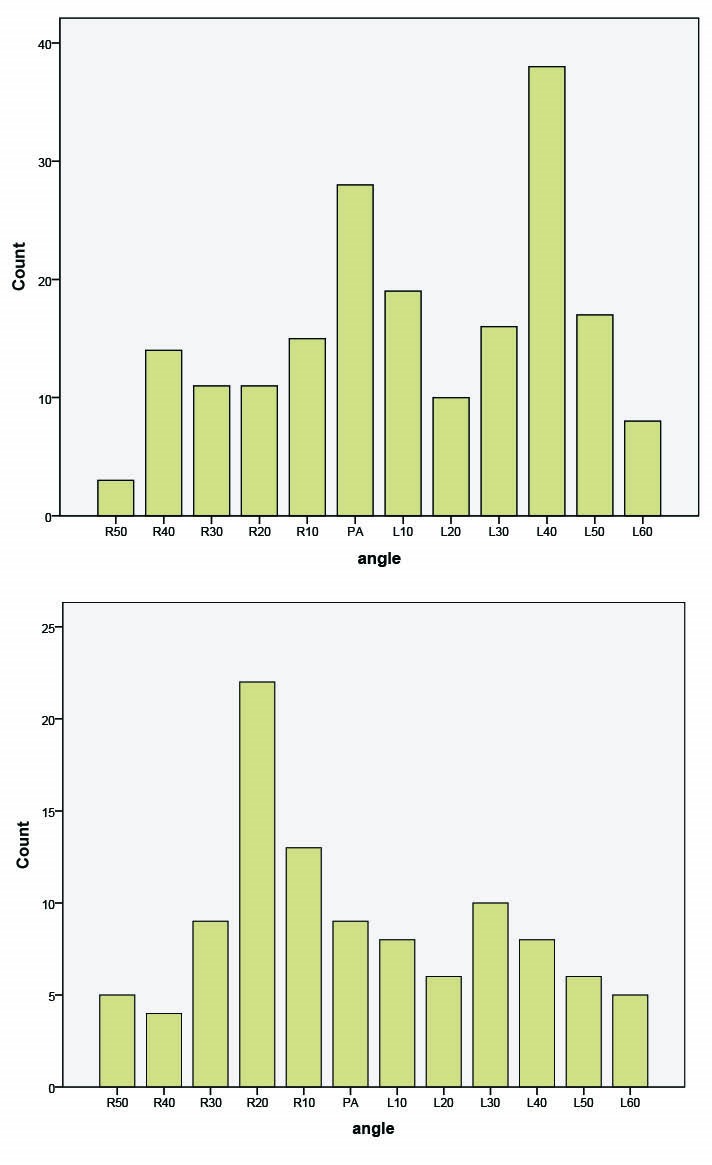
The frequency of projections in different angulations in Left-Anterior-Oblique (LAO) - Righr-Anterior-Oblique (RAO) and Posterioanterior (PA) projections. (A) Represents the frequencies in Coronary-Angiography. (B) Represent the frequencies in Percutaneous Coronary Intervention

The mean DAP for CAG was 436.73±315.85 dGy×cm^2^.Among the used projections in our study sample, mean DAP was lowest at 40° LAO/40° CR (32.7 dGy×cm^2^), and it was highest at 40° LAO/0° (1047 dGy×cm^2^) (Table 1). By comparing PA, CR and CA projections, it was found that posterioanterior (PA) projection had the greatest mean DAP (134±370.08 dGy×cm^2^) (Table 1). In addition; mean DAP was higher in caudal projection than CR projection (115.17±126.6 dGy×cm^2^and 68.95± 57.87 dGy×cm^2^, respectively). The projection 40 °LAO was the most frequently used projection in CAG (Figure 1). The projection 40° LAO/10° cranial and 40° LAO/10° caudal had 7 and 14 times less mean DAP compared to 40° LAO/0, respectively.

**Table 1:** Mean DAP in diagnosis coronary angiography in different angulations in unit of dGy×cm^ 2
^

	**Degree**	**RAO**					**PA**			**LAO**				
		**60** **51-60**	**50** **41-50**	**40** **31-40**	**30** **21-30**	**20** **11-20**	**10** **1-10**	**0**	**10** **1-10**	**20** **11-20**	**30** **21-30**	**40** **31-40**	**50** **41-50**	**60** **51-60**
CR	50 41-50										94				
	40 31-40							123	35	36.7					
	30 21-30			66.3	54.3			66.9	47	70.3	44	50	32.7	46	59
	20 11-20					54				26			73.3	92.7	166
	10 1-10				75			181		49.5		104	120		
PA	0								59.1				1047		
	10 1-10					38.2	110	141				161	157		35
	20 11-20						57						166		
	30 21-30					42.2	36.1	38						76	112
	40 31-40							99.5		115		106		103	301
CA	50 41-50							69			80			198	133

LAO: Left Anterior Oblique RAO: Right Anterior Oblique PA: Posterio Anterior CR: Cranial CA: Caudal

### Mean DAP in PCI

The mean DAP in PCI was 643.26±359.58 dGy×cm^2^. Among the projections that were used in our study sample, the projection 20° RAO/20° CR had the lowest mean DAP (9 dGy×cm^2^) and 40° RAO/30° CA had the highest mean DAP (746 dGy×cm^2^) (Table 2). When we compared PA, CR and CA projections, it was found that mean DAP was highest in CA (113.34±94.36 dGy×cm^2^) and it was higher in CR projection than PA projection (203.5±191.42 dGy×cm^2^ and 25.6±8.53 dGy×cm^2^, respectively). 20° RAO was the most common frequently used projection in PCI (Figure 1).

**Table 2:** Mean DAP in percutaneous coronary intervention in units of dGy×cm^2^

	**Degree**	**RAO**					**PA**			**LAO**				
		**60** **51-60**	**50** **41-50**	**40** **31-40**	**30** **21-30**	**20** **11-20**	**10** **1-10**	**0**	**10** **1-10**	**20** **11-20**	**30** **21-30**	**40** **31-40**	**50** **41-50**	**60** **51-60**
CR	50 41-50					41			777					
	40 31-40			320	242	176.33	197.2							
	30 21-30		108.2	62	38	77	106		35	86	116		230.33	
	20 11-20					9		10		26.5		46	77	47
	10 1-10										29			
PA	0							46						
	10 1-10						206		88		323.33	303		65
	20 11-20				148.5	15	91		99.66					
	30 21-30				309	200.55	57						98	245
	40 31-40				749	216	169.5		132		141	622		212.5
CA	50 41-50				61	212.66	179.75		101	213.33		139		

LAO: Left Anterior Oblique RAO: Right Anterior Oblique PA: Posterio Anterior CR: Cranial CA: Caudal

### Mean DAP Rate

### Mean DAP rate in CAG

By considering the time of employing each projection, DAP rate (DAP per second) was calculated in different angulations. The mean DAP rate was 3.13±3.1 dGy×cm^2^/sec. The projection 10° LAO/20° CR had the lowest DAP rate (0.09 dGy×cm^2^/sec), and 40° LAO/0° had the highest DAP rate (28.98 dGy×cm^2^/sec) (Table 3)

**Table 3:** Mean DAP rate in diagnosis coronary angiography in different angulations in unit of dGy×cm^ 2
^

	**Degree**	**RAO**					**PA**			**LAO**				
		**60** **51-60**	**50** **41-50**	**40** **31-40**	**30** **21-30**	**20** **11-20**	**10** **1-10**	**0**	**10** **1-10**	**20** **11-20**	**30** **21-30**	**40** **31-40**	**50** **41-50**	**60** **51-60**
CR	50 41-50									8.54				
	40 31-40						4.24	8.75	12.22					
	30 21-30		5.19	4.18			7.74	4.27	4.68	18.91	10	3.36	5.61	11.8
	20 11-20				6.75				0.09			14.02	10.11	6.38
	10 1-10			16.62			1.81		1.23		2.73	2.78		
PA	0							2.38				28.98		
	10 1-10				4.59	6.11	1.68	1.38			1.75	4.19		1.59
	20 11-20					2.11						5.75		
	30 21-30				2.02	2.16	1.36						7.92	22.87
	40 31-40						8.35		14.37		6.56		7.68	8.25
CA	50 41-50						9.86			7.9			14.14	3.8

LAO: Left Anterior Oblique RAO: Right Anterior Oblique PA: Posterio Anterior CR: Cranial CA: Caudal

By comparing the PA, CR and CA projections, DAP rate was highest in CR projection (7.02±7.1 dGy×cm^2^/sec) and caudal projection had higher DAP rate than PA projection (5.2±5.2 dGy×cm^2^/sec and 4.3±11.53 dGy×cm^2^/sec, respectively).

### Mean DAP rate in PCI

The mean DAP rate was 3.53±11.28 dGy×cm^2^/sec. Among the projections, which were used during PCI, DAP rate was lowest in 10° RAO/30° CR (0.5 dGy×cm^2^/sec), and it was highest in 20°RAO/30° CR (29.83 dGy×cm^2^/sec) (Table 4). Moreover, this projection (20° RAO/30° CR) was the most common used one.

**Table 4:** Mean DAP rate in in percutaneous coronary intervention in units of dGy×cm^2^

	**Degree**	**RAO**					**PA**			**LAO**				
		**60** **51-60**	**50** **41-50**	**40** **31-40**	**30** **21-30**	**20** **11-20**	**10** **1-10**	**0**	**10** **1-10**	**20** **11-20**	**30** **21-30**	**40** **31-40**	**50** **41-50**	**60** **51-60**
CR	50 41-50					4.55								
	40 31-40			0.76	0.86	0.69	3.15							
	30 21-30		1.83	3.87	2.11	29.83	0.5		0.85	3.74	1.27		2.43	
	20 11-20					2.25		0.63		3.13		3.54	2.75	3.62
	10 1-10										0.97			
PA	0							0.71						
	10 1-10						0.93		1.05		1.56	1.07		3.82
	20 11-20				1.17	0.83	5.35		2.18					
	30 21-30				1.17	1.21	0.71						1.69	4.54
	40 31-40				2.7	1.24	0.9		1.27		2.14	1.5		2.89
CA	50 41-50				1.56	2.93	2.09		4.34	2.95		1.85		

LAO: Left Anterior Oblique RAO: Right Anterior Oblique PA: Posterio Anterior CR: Cranial CA: Caudal

When we compared PA, CR and CU projections, DAP rate was highest in CR projection (3.58±7.87 dGy×cm^2^/sec) and it was higher in CA than PA projection (1.19±1.51 dGy×cm^2^/sec and 0.67±0.27 dGy×cm^2^/sec, respectively).

DAP rate was about 14 times higher in this projection, compared to 20 RAO/20 CA and it was about 42 times higher compared to 20 RAO/40 CR.

## Discussion

This study highlights the impact of selecting different sets of projections in CAG and PCI on patients’ radiation exposure. The effect of Ionizing radiation on patients’ health is a main concern, and this issue encourages researchers and cardiologists to identify and employ the projections that offer an excellent look with the minimal radiation dose. Equipment and even the cardiologist’s expertise influence the patient’s radiation exposure.^11-13^ Considering this fact, in the present study, only procedures performed by a single cardiologist on the same angiography unit were evaluated. The mean DAP of diagnostic CAG was 436.73±315.85 dGy×cm^2^. This finding is in consistent with the published work of Morrish and Goldstone in which the mean DAP value of different studies were calculated and reported to be 49.9±22.5 Gy×cm^2^ (499±225 dGy×cm^2^). Mean DAP of PCI in our study was 643.26±359.58 dGy×cm^2^ which is less than the reported mean DAP of 815 dGy×cm^2^ by Bedetti et al.^5^ On the other hand, it is higher than the mean DAP of 400 dGy×cm^2^, presented in a study by Vano et al.^8^ The complexity of the procedure, body mass index of patients and the experience of the performing cardiologist can affect the measured DAP^18^ and may explain some of the differences in various reports.

According to our results, the mean fluoroscopy time was 187±139 seconds for CAG, and it was 489±344 seconds for PCI. This finding is in consistent with the reported results by Kuon et al.^17^ Fluoroscopy time has been described as an influential factor on DAP. Georges et al.^19^, for example, evaluated DAP and fluoroscopy time during CAG and PCI and found their significant correlation. Moreover, Journy et al. examined contributing factors related to maximum skin dose (MSD) in interventional cardiology. In their study, MSD was significantly correlated to DAP in CAG but in PCI, other factors such as fluoroscopy time and body mass index were better independent predictors of MSD.^18^ Therefore, reduction in fluoroscopy time may prevent skin injuries in patients undergoing these procedures. Accurate diagnosis in CAG and performing PCI requires multiple views to observe all coronary segments clearly without foreshortening or overlapping the several views of choice are classically defined to offer the best possible look for the cardiologist. However, these projections may deliver higher radiation to the patient and even the cardiologist.^4^ In the present study, the measured DAP in each projection was divided by the fluoroscopy time in that projection and DAP rate was calculated to get a better understanding of radiation risk in each projection. In our study sample, the projection 40° LAO/0° accounted for the highest DAP rate in CAG compared to other projections. Moreover, it was the most frequently used projection in diagnostic CAG. This shows a need for changing projections to less irradiating ones when it is feasible. For instance, employing the projection 40° LAO/10° caudal could lower the DAP rate 14 times. On the other hand, the steep (≥40) LAO projections are reported to have greater scatter dose, and some studies discourage routine use of this projection.^17^ In this study the projection 20° RAO/30° CA had high levels of DAP rate. This projection is mainly used during the PCI of proximal left anterior descending artery. According to our results and based on patient safety concerns, the most suitable alternatives are LAO/CA angulations. However, in some circumstances there is a conflict between patient safety concerns and physician’s safety. For example, in our study during PCI, LAO/CA views had lower DAP compared with RAO/CR. Smith et al.^16^ defined a set of angiographic views that maximizes clinical information yield for minimum radiation risk According to their study, the preferred projections for left coronary artery which promise minimal radiation exposure to patients, are LAO/CR, AP-RAO/CA, RAO/CA and AP-RAO/CR. In the case of right coronary artery, the projections LAO/CR, RAO and AP-RAO/CR are described to be the best set. In this study, we also compared the DAP rate between PA, caudal and cranial views in both CAG and PCI. Although certain cranial and caudal angulated views may provide far better anatomic presentation of desired arteries but from the viewpoint of radiation exposure, the highest DAP rate in our study sample was in the cranial view. Moreover, DAP rate was higher in caudal view in comparison to PA view.

In our current study, the mean DAP in different projections was recorded without any intervention. Unlike many studies that were performed on designed phantoms, in our study the practicing cardiologist carried out the selection of the angulations and best views clinically. As shown in this study, some common angulations that are used clinically for achieving a good view of coronary arteries simultaneously expose patients to high radiation levels and the resulting scattered dose affects the radiation exposure of the practicing cardiologists as well. The mean DAP in our study was in the common range of reported values; however some of the most frequently used angulations were those with highest DAP rate. Consequently, using the alternative angulations for each coronary artery with lower DAP rate and decreasing the fluoroscopic time as much as possible, may protect the patients from unnecessary radiation exposure.

## Ethical issues

The research design was reviewed and approved by the Institutional Review Board Committee at Tabriz University of Medical Sciences.

## Competing interests

Authors declare no conflict of interest in this study.
